# Supplementary figures and images for: Rapid Aggregation of Staphylococcus aureus in Synovial Fluid Is Influenced by Synovial Fluid Concentration, Viscosity, and Fluid Dynamics, with Evidence of Polymer Bridging
Source: mBio. 2022 Mar 7;13(2):e00236-22. doi: 10.1128/mbio.00236-22 (PMC9040867; doi:10.1128/mbio.00236-22)

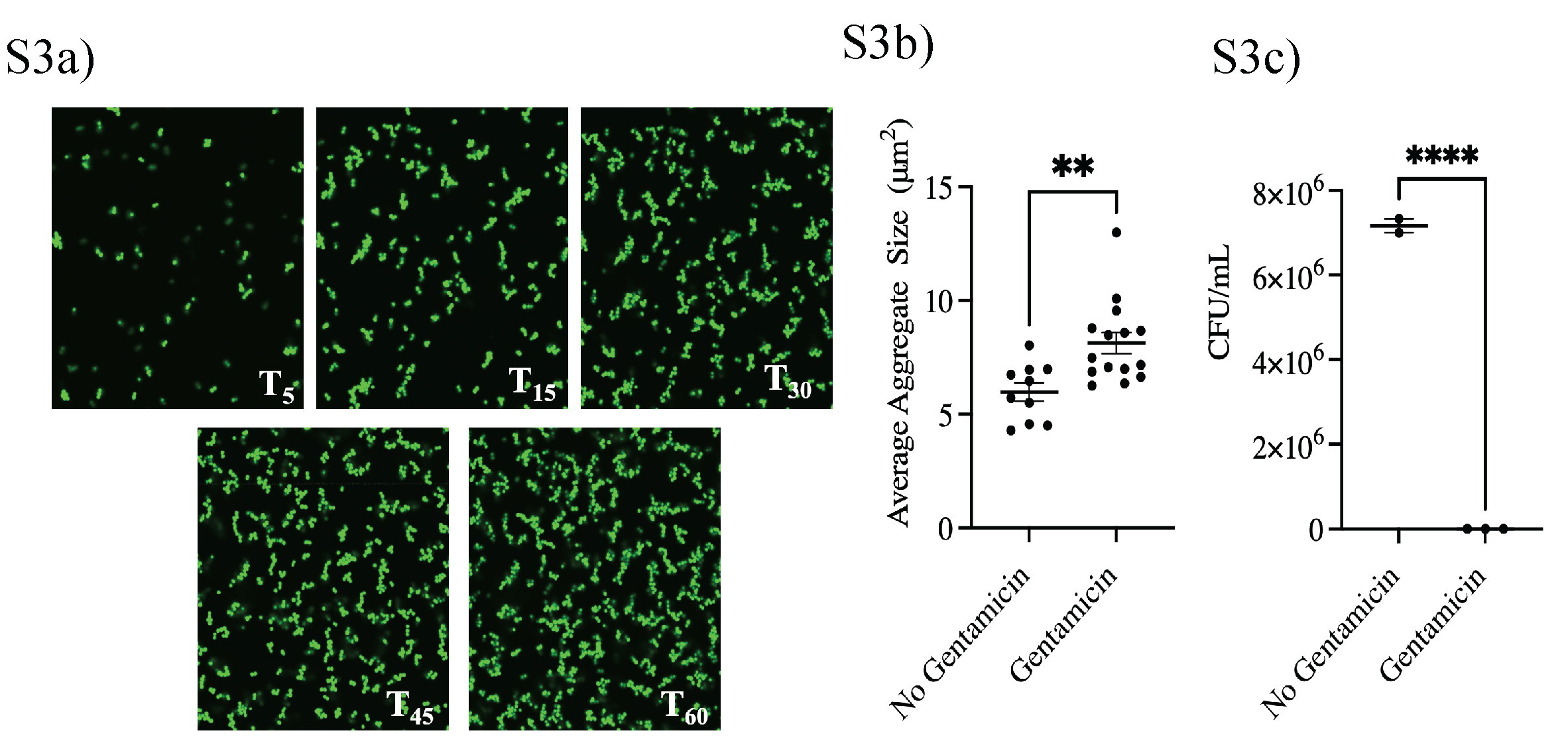

Supplement: FIG S3 [file mbio.00236-22-sf003.tif]

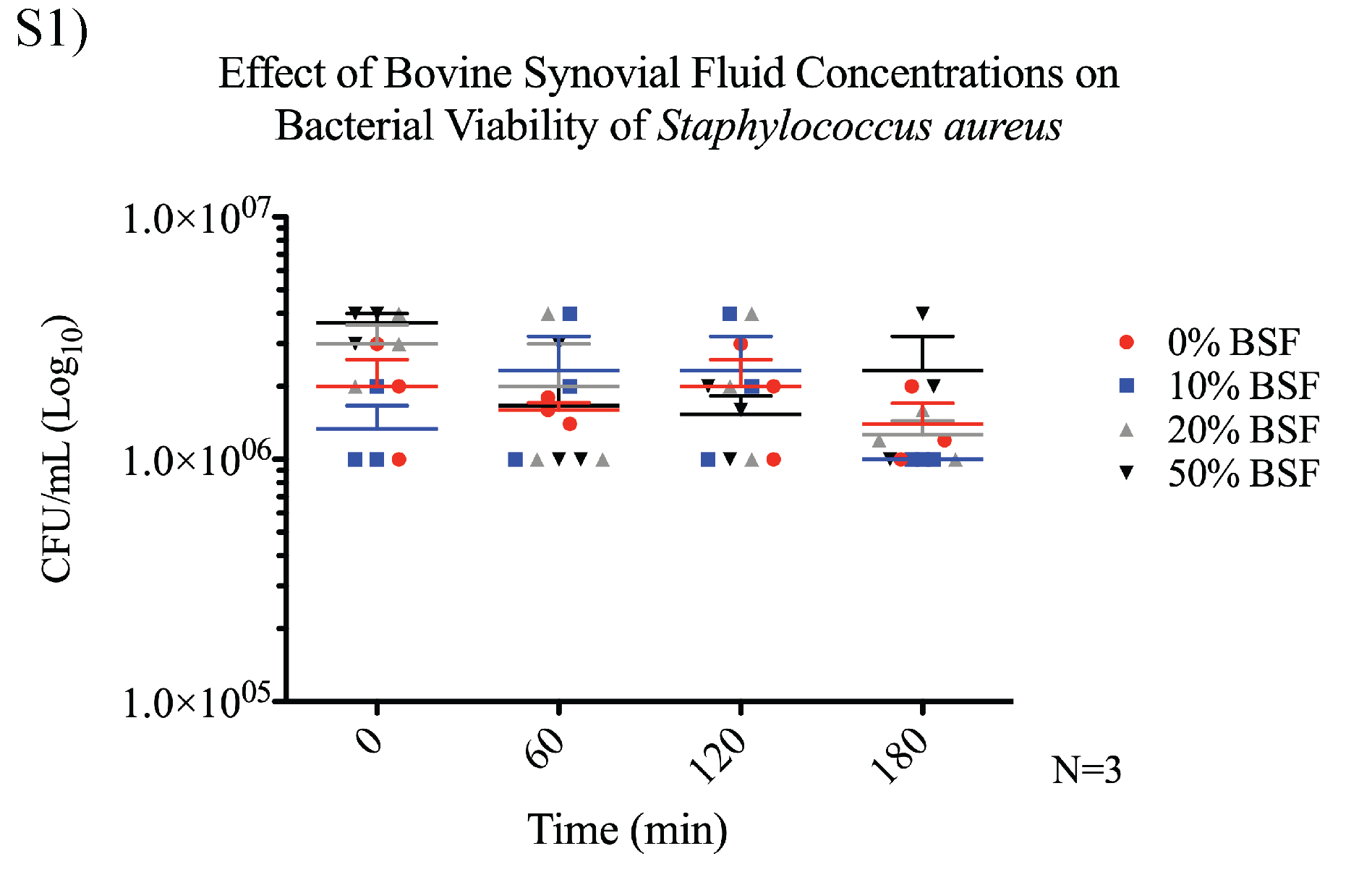

Supplement: FIG S1 [file mbio.00236-22-sf001.tif]

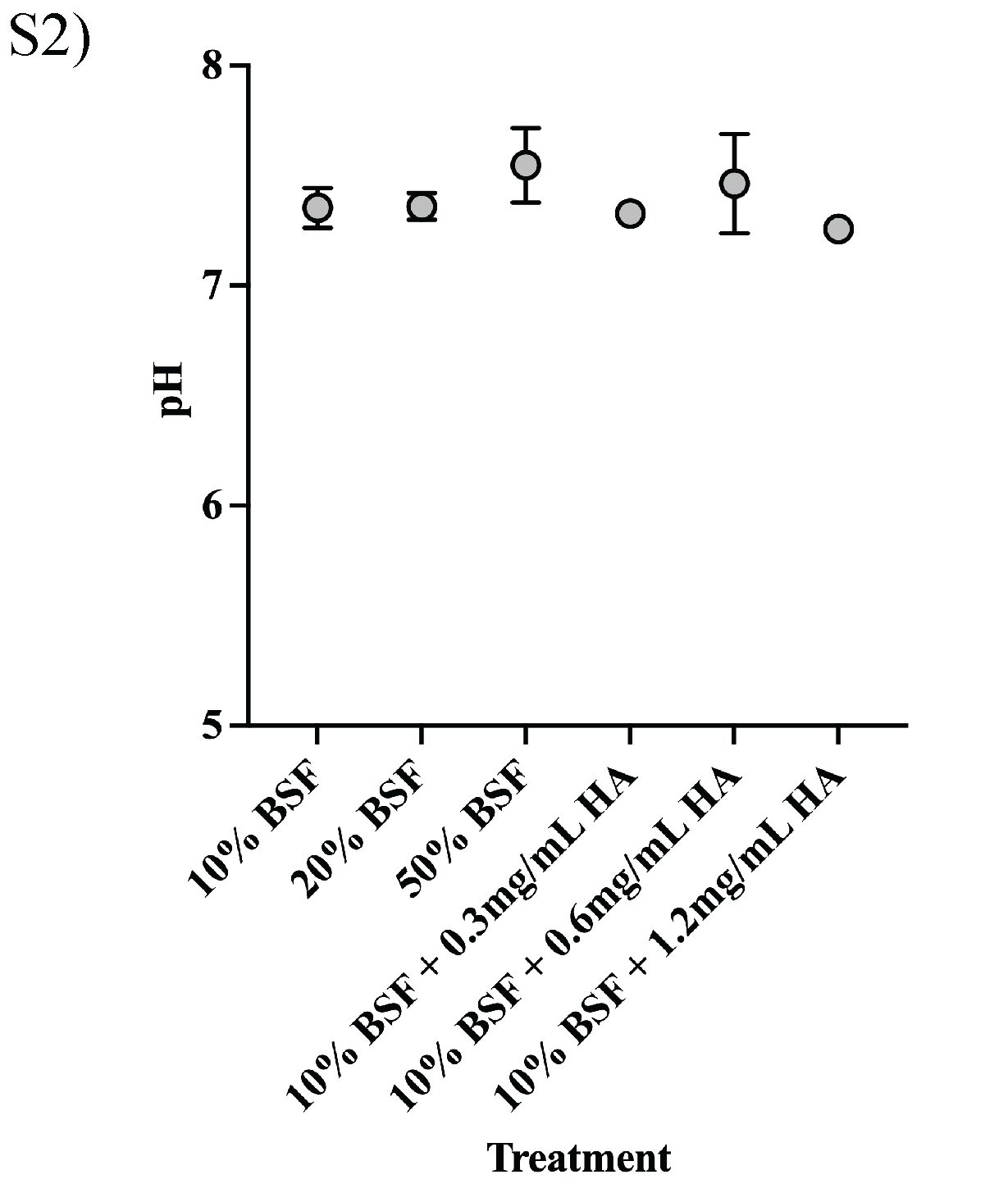

Supplement: FIG S2 [file mbio.00236-22-sf002.tif]
