# Supplementary material for: Rapid Aggregation of Staphylococcus aureus in Synovial Fluid Is Influenced by Synovial Fluid Concentration, Viscosity, and Fluid Dynamics, with Evidence of Polymer Bridging
Source: mBio. 2022 Mar 7;13(2):e00236-22. doi: 10.1128/mbio.00236-22 (PMC9040867; doi:10.1128/mbio.00236-22)
Supplement: TEXT S1 [file mbio.00236-22-t0001.docx]

**Supplemental Methods**

**Growth Curves**

Growth curves were conducted for AH1726 to assess the antimicrobial effects of synovial fluid within 3 hours (**Supplemental Figure 1**). A broth culture of AH1726 was grown overnight in TSB as previously described and then diluted 1:1000 into 5mL of phosphate-buffered saline (PBS) or supplemented bovine synovial fluid (BSF) (Lampire Biological Laboratories, Pipersville, PA, USA) in PBS. Dilutions were made in 10% BSF in PBS, 20% BSF in PBS, or 50% BSF in PBS. Following inoculation, 10µL were taken from each inoculum, serially diluted in PBS, and plated for colony-forming unit (CFU) counts on TSA plates. For growth curves, the inoculated tubes were incubated for 3-hours at 37°C in an orbital shaker (200 RPM) with sampling and CFU plating conducted every hour. Three biological replicates were conducted for each condition.

**Rheometer viscosity measurements**

The viscosity of synovial fluid-supplemented PBS, as well as hyaluronic acid-supplemented PBS, was determined using a rheometer (TA Instruments, New Castle, DE). 6mL samples of 10% BSF in PBS, 20% BSF in PBS, 50% PBS, as well as increasing concentrations of hyaluronic acid supplemented into 10% BSF, were prepared in 15mL falcon tubes. The rheometer was calibrated for a 3mm geometry before measurements were collected. The samples were vortexed and 2mL was slowly transferred between the stage and geometry at the loading gap (2,000µm). The geometry was then lowered to the trim gap (1,500µm) and the excess sample was removed with a Q-tip. After trimming, the geometry was lowered to the geometry gap (1,000µm) and a standard creep test was conducted to calculate the viscosity. The 5 viscosity readings immediately following the minimum viscosity, where readings were the most consistent, were obtained for each creep test and averaged for the final viscosity measurement.

**Synovial fluid-induced aggregation of gentamicin-killed bacteria**

To determine whether synovial fluid-induced aggregation requires bacteria to be living and active, we conducted aggregation assays with gentamicin-treated *S. aureus.* An overnight culture of AH1726 was diluted 1:100 into a new tube containing fresh TSB. The day culture was grown to an OD_600_ of 0.5 in a 37°C orbital shaker. The day culture bacteria were challenged with 100µg/mL of gentamicin (GoldBio, St Louis, MO). The antibiotic-containing culture was then placed in a 37°C incubator for 3 hours. Following incubation in the antibiotic, 1mL aliquots of the culture were transferred to microcentrifuge tubes, pelleted, and resuspended in 1mL of PBS. The bacteria were washed a total of three times to remove residual gentamicin then sampled for CFU plating. Plates were incubated overnight at 37°C to confirm bacteria were non-viable. Dead bacteria were exposed to 10% BSF for 1-hour and imaged using a confocal microscope as described above. Representative images were collected and analyzed with FIJI image analysis software to quantify average aggregate size. Data was imported to Prism and statistical significance was determined by Student’s T-test to compare mean aggregate size between treated and untreated bacteria.
